# The Joy Treatment

**Published:** 1919-01

**Authors:** 


### The Joy Treatment.

Physicians of repute say that they are finding "the joy treatment" an effective remedy for shell-shock. The idea is to give the patient such pleasant occupations and things to think about that he will forget and in time be healed, largely through forgetfulness, of the shell shock. Commenting upon this, the Galveston Tribune, says:

"Put down as miraculous, but coming to us with uncontrovertible backing are stories of cripples being cured and made to throw away their crutches by the announcement of some piece of joy-giving news. Every physician knows the marvelous effect upon a patient wrought by the arrival at the bedside of some loved one, and instances are not so rare of those who have been brought back from the edge of the grave by the receipt of a long-expected letter from some near relative. Joy medicine would not attract so much attention by its cures, it is believed, were it enlisted more frequently and believed in more sincerely.

"There could be no more fit time for giving joy medicine an opportunity for demonstration than just now while the influenza epidemic is exerting its greatest power. True, the physicians have recommended the open air and the sunshine, personal cleanliness and sensible eating, but why not tell the people to look up rather than down, think about the happier matters of life, to spend less time in talking about the sadness which has visited this neighbor or the other; it may not effect a cure in every instance, but no one is prepared to claim that it will accomplish no good at all.

"Joy medicine is not going to cure every malady to which the human frame is liable, but the little story alluded to has furnished a hint which ought not to be lost, and it is to be hoped that some one with a mind and a heart will follow the suggestion, for out of it might come a blessing to the race, even as it has been to some two thousand American soldiers who otherwise might even now be suffering a visitation which might have been extended through the entire course of their life."

---

Four million starving war victims in western Asia. Help them out February 3-10, the week of the Armenian Relief Campaign for the Southwest.

